# *Panax ginseng* abuse exhibits a pro‐inflammatory effect by activating the NF‐κB pathway

**DOI:** 10.1002/fsn3.3011

**Published:** 2022-09-03

**Authors:** Wenjun Deng, Hangxiu Liu, Lanping Guo, Yongzhong Liu, Zhaocheng Ma

**Affiliations:** ^1^ Key Laboratory of Horticultural Plant Biology (Ministry of Education), College of Horticulture and Forestry Huazhong Agricultural University Wuhan China; ^2^ National Resource Center for Chinese Materia Medica China Academy of Chinese Medical Sciences Beijing China

**Keywords:** ginseng abuse syndrome, NF‐кB, *Panax ginseng*, pro‐inflammatory effect

## Abstract

*P. ginseng* (*Panax ginseng* C. A. Meyer) is a well‐known traditional medicine that has been used for thousands of years to treat diseases. However, “ginseng abuse syndrome” (GAS) often occurs due to an inappropriate use such as high‐dose or long‐term usage of ginseng; information about what causes GAS and how GAS occurs is still lacking. In this study, the critical components that potentially caused GAS were screened through a step‐by‐step separation strategy, the pro‐inflammatory effects of different extracts on messenger RNA (mRNA) or protein expression levels were evaluated in RAW 264.7 macrophages through quantitative real‐time polymerase chain reaction (qRT‐PCR) or Western blot, respectively. It was found that high‐molecular water‐soluble substances (HWSS) significantly increased the expression of cytokines (cyclooxygenase‐2 (COX‐2), inducible nitric oxide synthase (iNOS), and interleukin 6 (IL‐6)) and cyclooxygenase 2 (COX‐2) protein; gel filtration chromatography fraction 1 (GFC‐F1) further purified from HWSS showed prominent pro‐inflammatory effects by increasing the transcription of cytokines (COX‐2, iNOS, tumor necrosis factor alpha (TNF‐α), and interleukin 1β (IL‐1β)) as well as the expression of COX‐2 and iNOS protein. Moreover, GFC‐F1 activated nuclear factor‐kappa B (NF‐кB) (p65 and inhibitor of nuclear factor‐kappa B alpha (IκB‐α)) and the p38/MAPK (mitogen‐activated protein kinase) signaling pathways. On the other hand, the inhibitor of the NF‐κB pathway (pyrrolidine dithiocarbamate (PDTC)) reduced GFC‐F1‐induced nitric oxide (NO) production, while the inhibitors of the MAPK pathways did not. Taken together, GFC‐F1 is the potential composition that caused GAS through the production of inflammatory cytokines by activating the NF‐кB pathway.

## Graphical abstract

Excessive consumption of panax ginseng may cause over‐activation of the immune system forming "ginseng abuse syndrome". The present results suggested that GFC‐F1 purified from ginseng is the crucial component that caused "ginseng abuse syndrome". Moreover, GFC‐F1 activates the NF‐κB pathway to trigger the immune system leading to inflammatory responses through inducing the phosphorylation of p65 and IκBα.

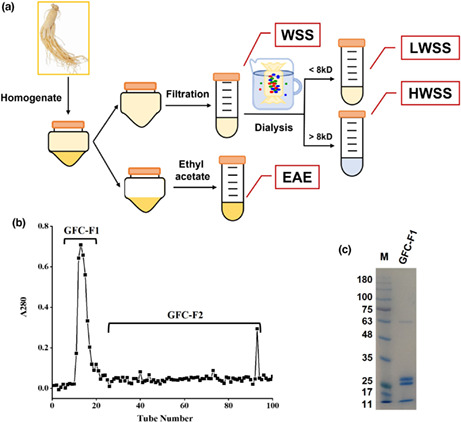

## 1 INTRODUCTION

Chinese herbal medicine has been used for preventing, treating, or curing diseases for thousands of years (Zhang et al., 2020). As the “king of herbs,” *P. ginseng* (*Panax ginseng* C. A. Meyer) has exerted multiple pharmacological effects, such as anti‐inflammatory, anti‐oxidation, antitumor, antidiabetes effects, and cardiovascular protection (Ichikawa et al., 2009; Jee et al., 2014; Ru et al., 2015; Yu et al., 2017). Therefore, it is always used as a therapeutic supplement or functional food to improve human health, including restoring physical vitality, enhancing immunity, and reducing cancer risk (Aminifard et al., 2021; Cho et al., 2014; Lee & Son, 2011). However, ginseng abuse or misuse, namely, long‐term or high‐dose consumption of ginseng, always occurred, which usually causes “*shanghuo*” (called in traditional Chinese medicine) (Xu & Dou, 2016), or “ginseng abuse syndrome” (GAS) (Siegel, 1979). GAS‐associated symptoms include hypertension, morning diarrhea, skin eruption, nervousness, and sleeplessness (Chan et al., 2011; Paik & Lee, 2015; Vazquez & Aguera Ortiz, 2002), similar to inflammation.

To date, the pharmacological functions of ginseng are attributed to abundant pharmacologically active compounds, such as ginsenosides, polysaccharides, peptides, polyacetylene alcohols, fatty acids, and proteins (Cho et al., 2014; Kim et al., 2017; Ru et al., 2015). Many studies have been focused on the mechanisms for the pharmacological role of such active compounds. For example, ginsenosides exerting antitumor effect are attributed to inhibit tumor invasion or metastasis and induce tumor cell apoptosis; their anti‐inflammatory effect takes place through inhibition of 12‐O‐tetradecanoylphorbol‐13‐acetate (TPA)‐induced COX‐2 expression (Vazquez & Aguera Ortiz, 2002) or the production of pro‐inflammatory cytokines by regulating the transcription factor NF‐κB and activator protein‐1 (AP‐1) (Jee et al., 2014); on the other hand, polysaccharides also play indispensable roles in the medicinal value of ginseng, such as enhancing immunity (Yu et al., 2014), regulating blood glucose levels (Ratan et al., 2021), exerting antioxidant effects (Kim et al., 2020), and causing antitumor effects (Jee et al., 2014), possibly by activating transcription factors (NF‐κB and AP‐1) along with their upstream signal transduction enzymes such as extracellular signal‐regulated kinase (ERK) and c‐Jun N‐terminal kinase (JNK) (Byeon et al., 2012). Although ginseng abuse or misuse always occurs in daily life, the underlying reason for producing GAS is almost ignored.

Inflammation is an innate immune response marked by capillary dilatation, leukocytic infiltration, redness, heat, and pain, and it can eliminate toxic stimuli and damaged tissues (Kim et al., 2017). As the primary immune cells for innate defense against infections (Kang & Min, 2012), macrophages play a crucial role in the inflammatory response. First, macrophages recognize harmful stimuli through toll‐like receptors (TLRs) and leucine‐rich repetitive sequence receptors (nucleotide oligomerization domain (NOD)‐like receptors (NLRs)) (Kim et al., 2017). Subsequently, these receptors trigger a series of signaling cascades, such as NF‐кB and MAPK pathways; NF‐кB is a crucial transcription factor connected to immune and acute inflammatory responses. The MAPK pathway, which activates downstream transcription factors that cause the transcription of pro‐inflammatory genes and the secretion of pro‐inflammatory cytokines (such as TNF‐α, IL‐1β, and IL‐6), is intimately tied to the activation of NF‐кB pathway (Shin et al., 2018). The transcriptional expression of inducible nitric oxide synthase (iNOS) will promote the persistent production of NO. COX‐2 is also an inducible enzyme and associated to the pathogenesis of inflammatory‐related diseases (Bai et al., 2019). However, the uncontrolled production of pro‐inflammatory mediators will evoke an inflammatory response (Azike et al., 2015; Deng et al., 2020). The continuous inflammatory reaction will lead to the overactivation of the innate immune system, in turn causing harmful pathological consequences to the host (García‐Lafuente et al., 2009).

Due to excessive consumption of *P. ginseng* inducing inflammation‐like symptoms, whether or not the causal mechanism for GAS involves the occurring process of inflammation and how to make such GAS are still unclear. This study aims to explore the bioactive components that caused GAS and reveal the underlying mechanism for producing GAS. In this study, we extracted different fractions from *P. ginseng* roots through an activity‐directed separation strategy and investigated their possible effects in vitro. Results showed that it is the water‐soluble fraction, GFC‐F1 (gel filtration chromatography fraction 1), from high‐molecular water‐soluble substances (HWSS) of *P. ginseng* that significantly induce the levels of pro‐inflammatory cytokines by activating NF‐κB, not the MAPK signaling pathway.

## 2 MATERIAL AND METHODS

### 2.1 Material and treatments

**FIGURE 1:**
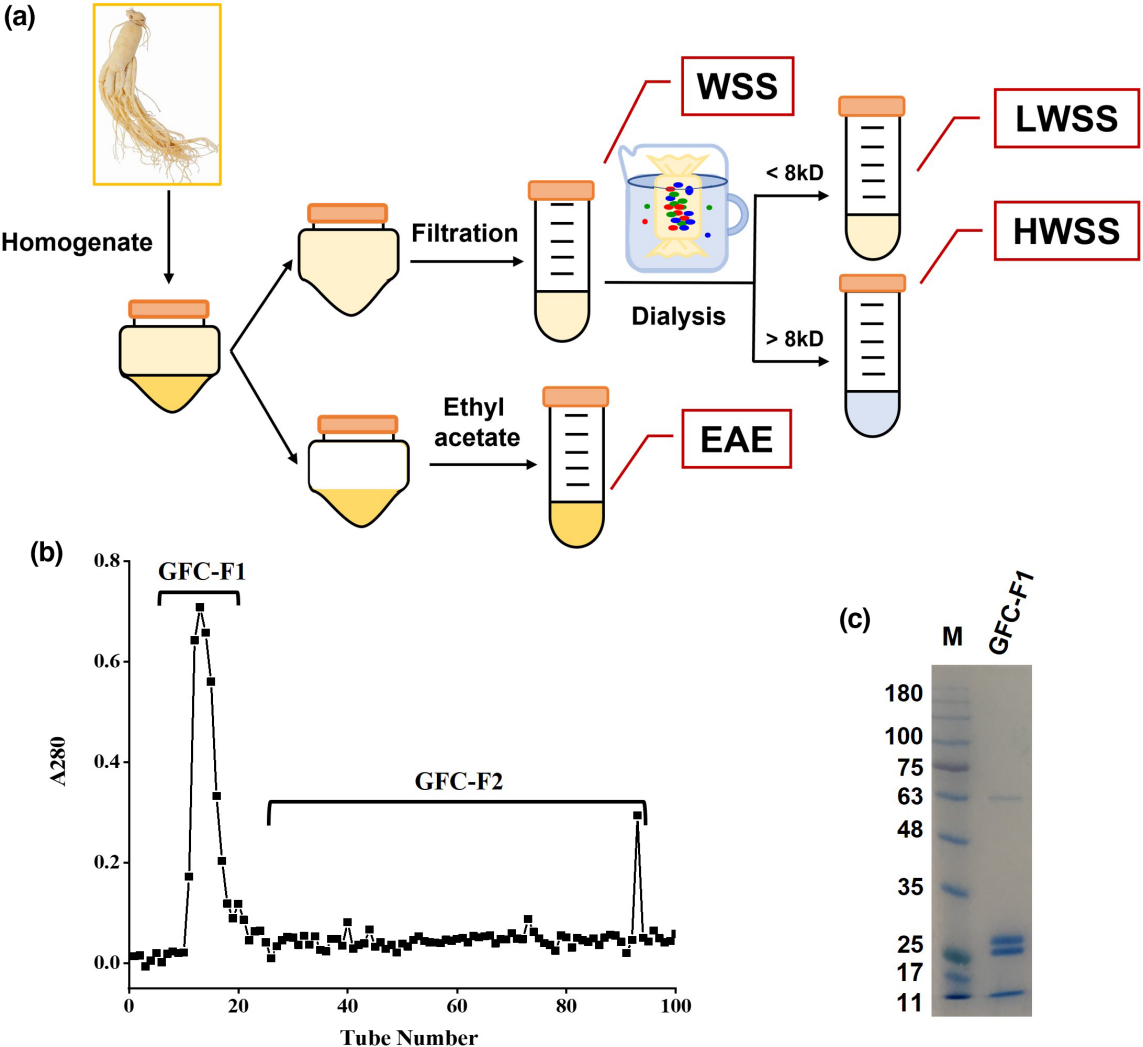
Screening flow of pro‐inflammatory components from *Panax ginseng* roots. (a) Isolation of LWSS and HWSS from ginseng roots; (b) purification of GFC‐F1 by GFC, the chromatogram of the eluent at 280 nm; (c) SDS‐PAGE analysis of GFC‐F1. EAE, ethyl acetate extracts; GFC, gel filtration chromatography; GFC‐F1, GFC fraction 1; GFC‐F2, GFC fraction 2; HWSS, high‐molecular water‐soluble substances; LWSS, low‐molecular water‐soluble substances; SDS‐PAGE, sodium dodecyl sulfate‐polyacrylamide gel electrophoresis; WSS, water‐soluble substances.

Fresh six‐year‐old *P. ginseng* roots were collected from the ginseng planting research base in Jilin province. First, the pro‐inflammatory components were screened from ginseng roots using a stepwise screening method (Yan et al., 2014). As shown in Figure 1a, 1 kg of ginseng roots was rinsed, dried, and added with 2 L of ultrapure water for homogenization. Next, the ginseng homogenate was centrifuged, and the resulting supernatant and precipitate were used to prepare water‐soluble substances (WSS) and ethyl acetate extracts (EAE), respectively. Then, WSS was separated into high‐molecular water‐soluble substances (HWSS) and low‐molecular compounds (LWSS) through dialysis with dialysis bags (8–14 kDa cut‐off) at 4°C for 3–5 days. The HWSS was further purified by gel filtration chromatography (GFC). After being filtered with a 0.22 μm microfiltration membrane (Millipore), the HWSS sample was loaded into a Sephadex G‐200 gel filtration column and eluted with 50 mM Tris–HCl buffer (pH 7.4, 0.5 mM ethylenediaminetetra acetic acid (EDTA), 50 mM NaCl) at a flow rate of 1 ml/min. All eluates were collected (3 min/tube). After detecting the ultraviolet (UV) absorbance at 280 nm, two fractions, GFC‐F1 and GFC‐F2 (Figure 1b), were obtained for further use.

### 2.2 Cell culture and cell viability assay

RAW 264.7 macrophages (Procell) were cultured in Dulbecco's modified Eagle's medium (DMEM) containing 10% fetal bovine serum (FBS), penicillin (100 U/ml), and streptomycin (100 μg/ml) at 37°C and 5% CO_2_ under humidified conditions in a cell incubator. When the cell density reaches 80%, it is harvested and transferred to a cell culture plate for treatment. The RAW 264.7 cells were treated with EAE/WSS (250, 500, 1000 μg/ml), LWSS/HWSS (0, 100, 200, 500, 1000 μg/ml), F1/F2 (0, 25, 50, 100, 200 μg/ml), or LPS (1 μg/ml) for 18 h, respectively.

Cell viability was performed to determine the growth inhibitory effects of ginseng extracts on RAW 264.7 cells at different concentrations with a cell counting kit‐8 (CCK‐8) testing kit (Zoman). In brief, the cells were cultured in a 96‐well plate at a density of 1 × 10^4^ cells/well. After 24‐h preincubation, the cells were treated with different ginseng extracts for 18 h, respectively. Subsequently, 10 μl of CCK‐8 solution was added to each well, and the cells were further incubated for 2 h. Finally, the absorbance was determined at 450 nm using a microplate reader (Tecan M200 PRO). All experiments were performed with three replicates.

### 2.3 RAW 264.7 cell treatments and quantitative real‐time PCR (qRT‐PCR) analysis

Total RNA was extracted from the cells by TRI‐pure reagent (Aidlab), and the first‐strand complementary DNA (cDNA) was synthesized using a reverse transcription kit (Aidlab) for the transcript analysis of pro‐inflammatory cytokine genes (*COX‐2*, *iNOS*, *TNF‐α*, *IL‐1β*, or *IL‐6*). The quantitative real‐time polymerase chain reaction (qRT‐PCR) was performed in a QuantStudio 6 Flex Real‐Time PCR Instrument (ABI) with the Hieff® qPCR SYBR Green Master Mix (Yeasen). The reactions followed the procedure of 50°C for 2 min, 95°C for 5 min; 40 cycles of 95°C for 15 s and 60°C for 30 s; and 72°C for 10 s. The *β‐actin* gene was used as an internal standard. The gene expression levels relative to the control were analyzed using the 2^−△△Ct^ method. The primers of each gene for qRT‐PCR are shown in Table 1.

**TABLE 1:** Primer sequences used in qRT‐PCR

Gene name	Forward/Reverse primer sequence (5′–3′)	References
*COX‐2*	ATCTGGCTTCGGGAGCACAAC	Cheng et al. (2017)
	GAGGCAATGCGGTTCTGATACTG	
*IL‐1β*	GTTGACGGACCCCAAAAGAT	
	CCTCATCCTGGAAGGTCCAC	
*IL‐6*	ACAAAGCCAGAGTCCTTCAGA	
	TCCTTAGCCACTCCTTCTGT	
*iNOS*	GAATCTTGGAGCGAGTTGTGGA	Guo et al. (2016)
	GTGAGGGCTTGGCTGAGTGAG	
*TNF‐α*	CTTGTTGCCTCCTCTTTTGCTTA	
	CTTTATTTCTCTCAATGACCCGTAG	
*β‐Actin*	AGGCTGTGCTGTCCCTGTATGC	Lee et al. (2020)
	ACCCAAGAAGGAAGGCTGGAAA	

Abbreviations: COX‐2, cyclooxygenase‐2; IL‐1β, interleukin 1β; IL‐6, interleukin‐6; iNOS, inducible nitric oxide synthase; TNF‐α, tumor necrosis factor α.

### 2.4 Nitric oxide (NO) assay

After 18‐h cell incubation with samples of different concentrations, the nitrite (sodium nitrite (NaNO_2_)) content was quantified using a Total Nitric Oxide Assay Kit (Beyotime, Shanghai, China). Briefly, 50 μl of culture supernatants was mixed with 50 μl of Griess reagents І and II. The absorbance at 540 nm was measured. Nitric oxide (NO) concentrations were calculated with NaNO_2_ as the standard curve. In the inhibitor experiment, the cells were pretreated with inhibitors for 2 h before the sample treatments.

### 2.5 Western blot analysis

After 12‐h cell incubation with samples of different concentrations, the cells were washed with prechilled phosphate‐buffered saline (PBS) three times and lysed with lysis buffer. After centrifugation (12,000 g, 4°C, 5 min), the supernatant was collected for Western blot analysis. The proteins were quantified with a bicinchoninic acid assay (BCA) kit.

The proteins from different cell extracts were resolved by 10% sodium dodecyl sulfate‐polyacrylamide gel electrophoresis (SDS‐PAGE) and then transferred onto polyvinylidene fluoride (PVDF) membranes (Bio‐Rad). These membranes were blocked with Tris‐buffered saline with Tween 20 (TBST) buffer containing 5% skimmed milk for 2 h at room temperature. The blocked membranes were then incubated overnight at 4°C with appropriate primary antibodies (p65/p‐p65, IκB‐α/p‐IκB‐α, p38/p‐p38, JNK/p‐JNK, and ERK/p‐ERK). The next day, these membranes were rinsed six times (each time for 5–10 min), followed by a 2‐h incubation with horseradish peroxidase (HRP)‐conjugated secondary antibody. Immunolabeling was detected using an ECL (Enhanced Chemiluminescence) Western blot kit (Bio‐Rad).

### 2.6 Statistical analysis

All results were analyzed with SPSS Statistics v.19 and shown as the mean ± standard error (SE). The statistical analysis was performed by one‐way analysis of variance (ANOVA) followed by Duncan's multiple range tests for comparison among different groups. The differences at *p* < .05 were considered to be statistically significant. The signaling pathway map was created with BioRender.com.

## 3 RESULTS

### 3.1 Effects of EAE and WSS on cell viability and pro‐inflammatory activity

**FIGURE 2:**
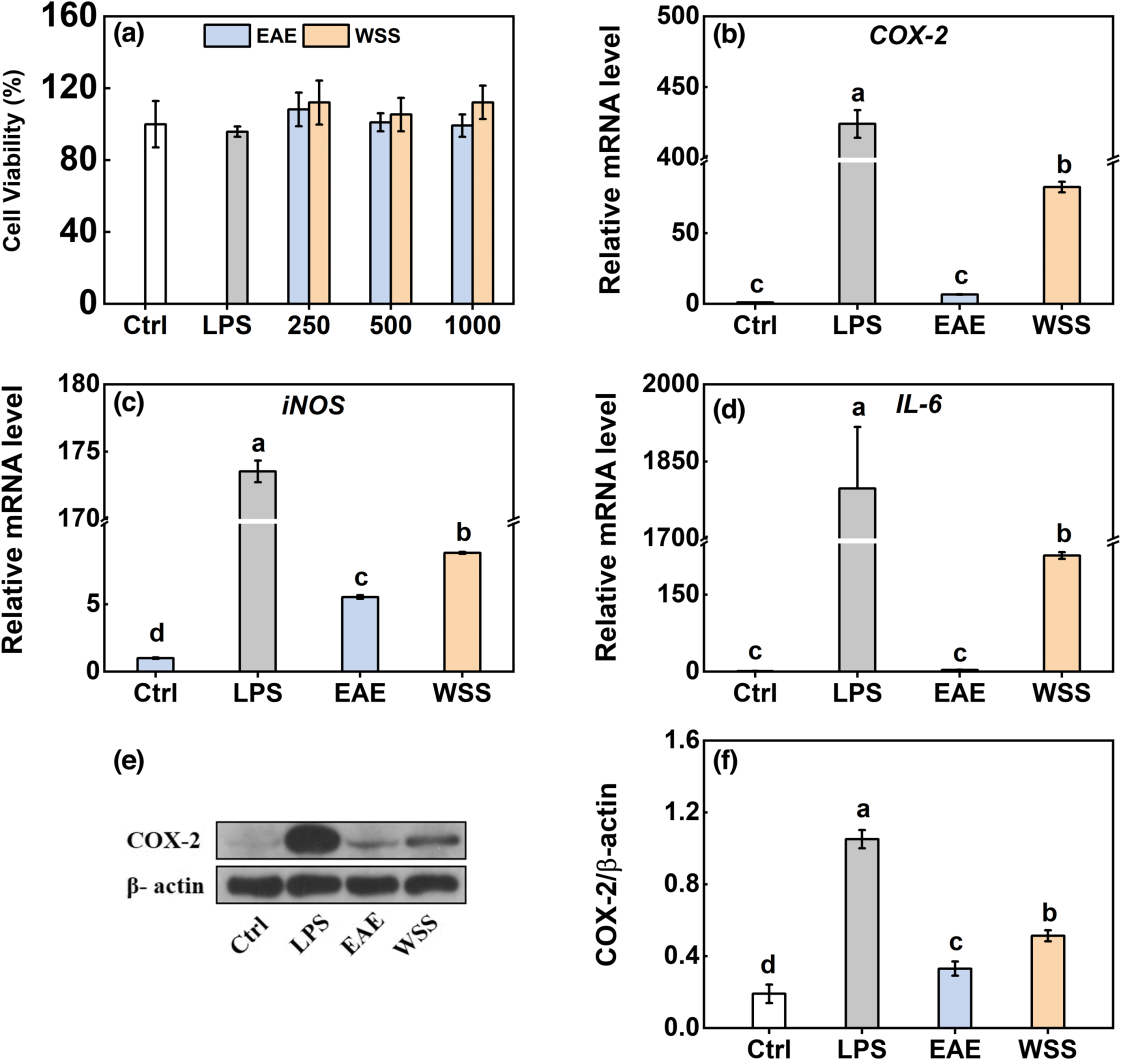
Effects of EAE and WSS on RAW 264.7 macrophages. (a) Effects of EAE and WSS on cell viability. The cells were treated with different concentrations of EAE and WSS (250, 500, and 1000 μg/ml) for 18 h. LPS (1 μg/ml) as a positive control. The viability was measured by a CCK assay kit. After the cells were treated with 1000 μg/ml of EAE/WSS for 18 h, the mRNA levels of *COX‐2* (b), *iNOS* (c), and *IL‐6* (d) were measured by qRT‐PCR. (e,f) The COX‐2 protein level was measured by Western blot analysis. Data are presented as the mean ± SE (*n* = 3), and different lowercase letters on the bar indicate significantly different values (*p* < .05). CCK, cell counting kit; COX‐2, cyclooxygenase‐2; EAE, ethyl acetate extracts; IL‐6, interleukin‐6; iNOS, inducible nitric oxide synthase; LPS, lipopolysaccharide; mRNA, messenger RNA; qRT‐PCR, quantitative real‐time polymerase chain reaction; SE, standard error; WSS, water‐soluble substances.

Cytotoxic effects of ginseng crude extracts (EAE and WSS) were initially investigated on RAW 264.7 macrophages through a CCK‐8 testing kit (Figure 2a). Similar to the lipopolysaccharide (LPS) treatment, EAE and WSS at different concentrations did not significantly change the cell viability of macrophages compared to the control. Both EAE and WSS did not cause any significant cytotoxicity in 264.7 cells, so subsequent experiments used the concentrations (1000 μg/ml), showing no cytotoxic effects. However, the pro‐inflammatory cytokines' gene levels were significantly lower in EAE‐ and WSS‐treated groups than those in the LPS‐treated group (Figure 2b–d). Moreover, the expression levels of *COX‐2*, *iNOS*, and *IL‐6* mRNA (messenger RNA) in the WSS‐treated group were 12.5‐, 1.6‐, and 67.4‐fold higher than those in the EAE‐treated group, respectively. Similar to gene expression, the COX‐2 protein level in the WSS‐treated group was lower than that in the LPS‐treated group but higher than that in the EAE‐treated group or control group (Figure 2e,f). By the way, only the iNOS mRNA level was significantly induced by EAE compared to the control (Figure 2c).

### 3.2 Effects of LWSS and HWSS on cell viability and pro‐inflammatory activity

**FIGURE 3:**
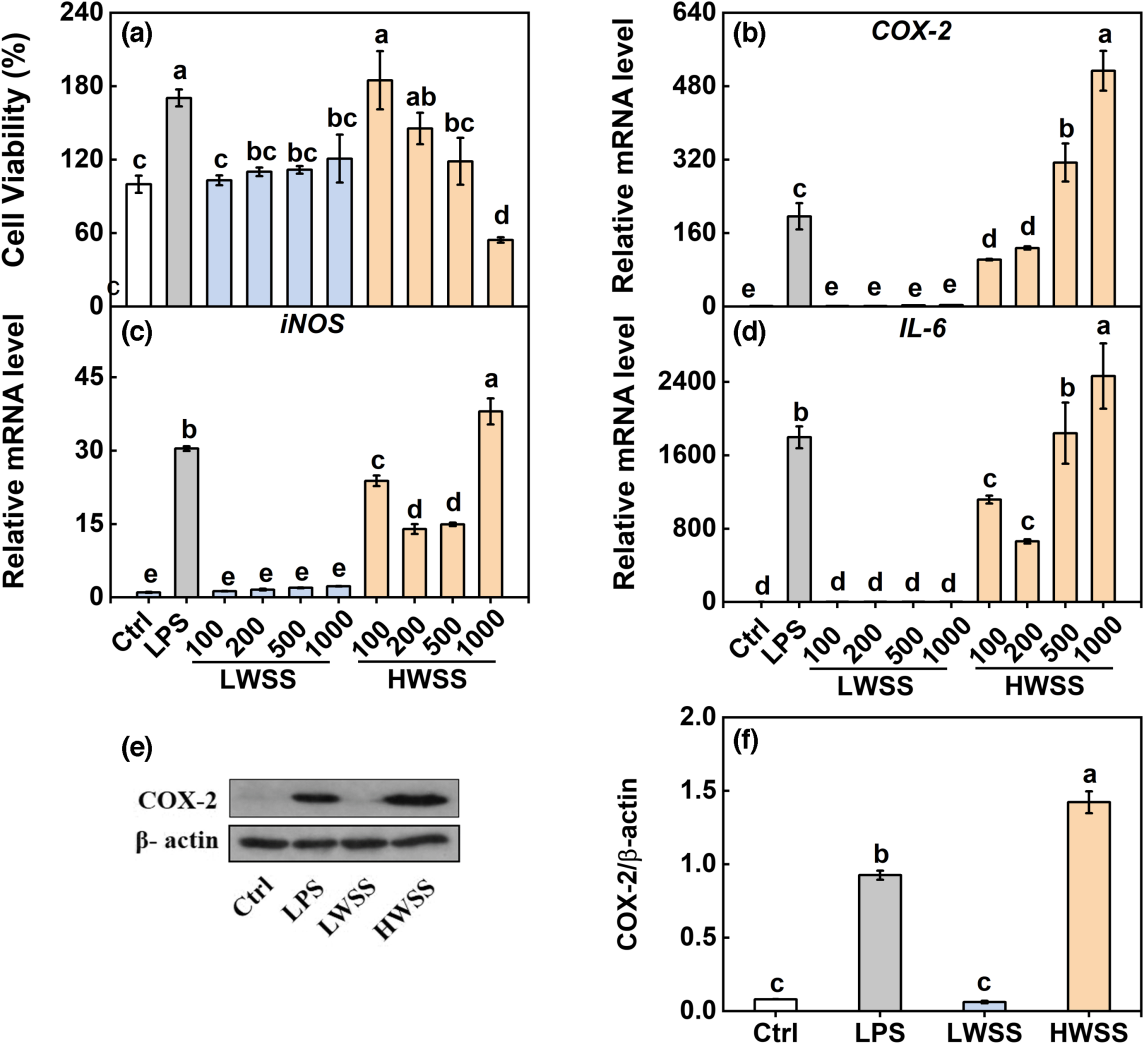
Effects of LWSS and HWSS on RAW 264.7 macrophages. Cells were treated with various concentrations of LWSS and HWSS (100, 200, 500, and 1000 μg/ml) for 18 h. LPS (1 μg/ml) as a positive control. (a) The viability was measured by a CCK assay kit. Cells were treated with 500 μg/ml of LWSS/HWSS for 18 h, and the mRNA levels of *COX‐2* (B), *iNOS* (C), and *IL‐6* (D) were measured by qRT‐PCR. (e,f) The COX‐2 protein level was measured by Western blot analysis. Data are presented as the mean ± SE (*n* = 3), and different lowercase letters on the bar indicate significantly different values (*p* < .05). CCK, cell counting kit; COX‐2, cyclooxygenase‐2; HWSS, high‐molecular water‐soluble substances; IL‐6, interleukin‐6; iNOS, inducible nitric oxide synthase; LPS, lipopolysaccharide; LWSS, low‐molecular water‐soluble substances; mRNA, messenger RNA; qRT‐PCR, quantitative real‐time polymerase chain reaction.

The WSS extract was further divided into two fractions (LWSS and HWSS) through dialysis (Figure 1a). Cell viability assay indicated no significant difference in cell viability between the control and LWSS‐treated group. However, the cell viability was significantly increased by the supplement of 100 or 200 μg/ml of HWSS, while it was significantly decreased by 1000 μg/ml of HWSS compared to the control. Moreover, the cell viability in the group treated by 100 or 200 μg/ml of HWSS was similar to that of the LPS‐treated group (Figure 3a).

The response of pro‐inflammatory cytokines' genes (*COX‐2, iNOS*, and *IL‐6*) was subsequently investigated (Figure 3b–d). The LPS did significantly induce the transcript levels of *COX‐2, iNOS*, and *IL‐6* compared to the control. Similarly, HWSS at different concentrations (100, 200, 500, and 1000 μg/ml) dramatically boosted the transcript levels of *COX‐2, iNOS*, and *IL‐6* compared to the control. On the other hand, no significant difference in such genes' mRNA levels was found between the control and the LWSS‐treated groups. In addition, the COX‐2 protein content was also markedly induced by HWSS, similar to the treatment of LPS, while the COX‐2 protein content in the LWSS‐treated group was similar to the control (Figure 3e–f).

### 3.3 Effects of GFC fractions on cell viability and pro‐inflammatory activity

**FIGURE 4:**
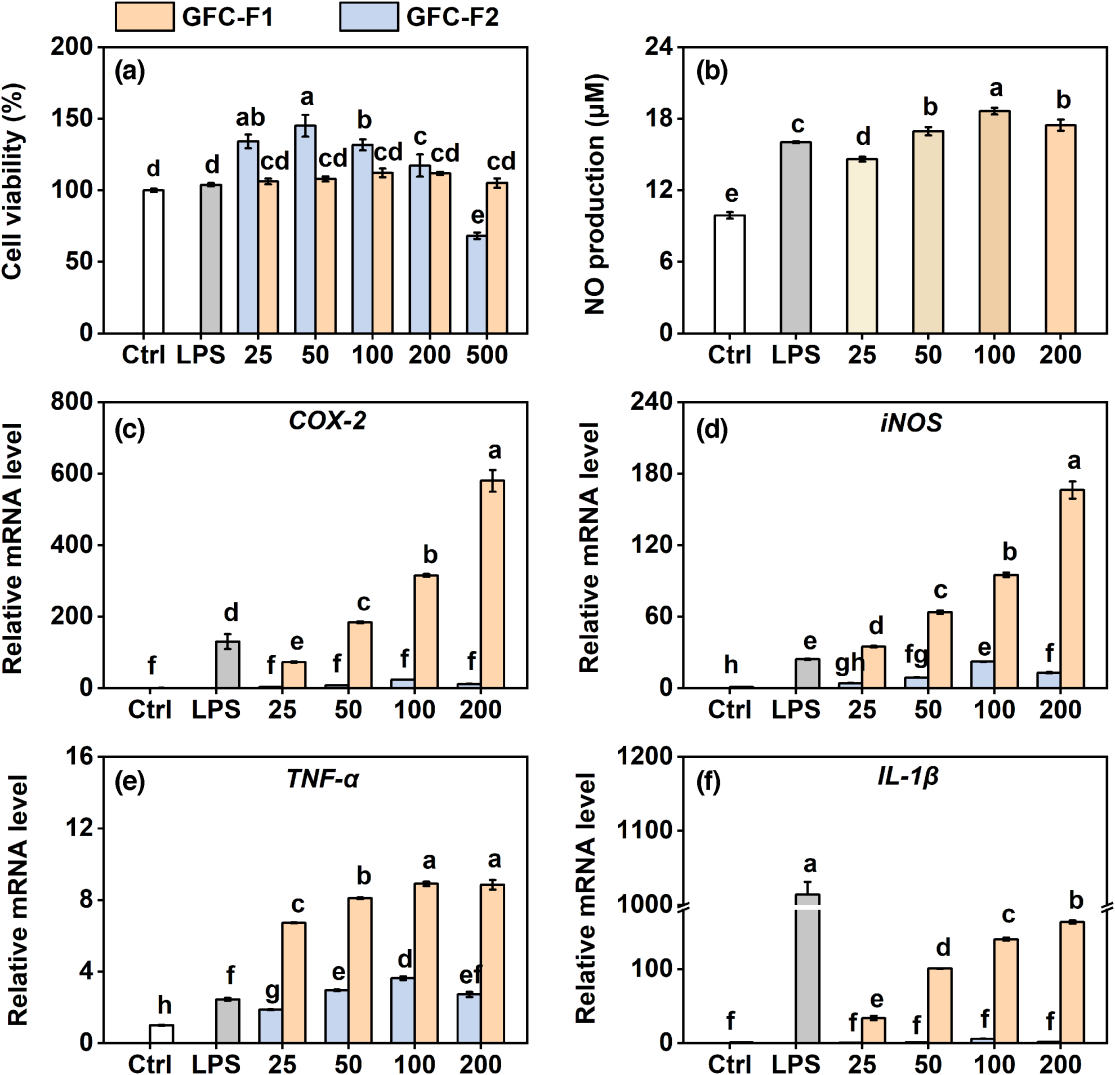
Effects of GFC fractions on RAW 264.7 macrophages. (a) Effects of GFC fractions on cell viability. Cells were treated with various concentrations of GFC‐F1/GFC‐F2 (25, 50, 100, 200, and 500 μg/ml) for 18 h. LPS (1 μg/ml) as a positive control. The cells were measured by a CCK assay kit. Cells were treated with various concentrations of GFC‐F1/GFC‐F2 (25, 50, 100, and 200 μg/ml) for 18 h. The NO production (b) was measured by the Griess assay. The mRNA expression of *COX‐2* (c), *iNOS* (d), *TNF‐α* (e), and *IL‐1β* (f) was measured by qRT‐PCR. Data are presented as the mean ± SE (*n* = 3), and different lowercase letters on the bar indicate significantly different values (*p* < .05). CCK, cell counting kit; COX‐2, cyclooxygenase‐2; GFC, gel filtration chromatography; GFC‐F1/2, gel filtration chromatography fraction 1/2; IL‐1β, interleukin 1β; iNOS, inducible nitric oxide synthase; LPS, lipopolysaccharide; mRNA, messenger RNA; NO, nitric oxide; qRT‐PCR, quantitative real‐time polymerase chain reaction; TNF‐α, tumor necrosis factor‐α.

The HWSS with pro‐inflammatory activity was further separated into two fractions (GFC‐F1 and GFC‐F2) by GFC (Figure 1b). The SDS‐PAGE analysis revealed that GFC‐F1 mainly contained four obvious band‐sized proteins with approximately 11, 27, 29, and 60 kDa, respectively (Figure 1c). However, no significant difference in cell viability was found between GFC‐F1‐treated and control or LPS‐treated groups. On the other hand, the cell viability of RAW 264.7 macrophages was significantly increased by the supplement of 25–200 μg/ml of GFC‐F2, while it declined considerably by the treatment of 500 μg/ml of GFC‐F2 compared to the control (Figure 4a). Moreover, GFC‐F1 also markedly increased NO production in the medium of RAW 264.7 cells; the NO yield was noticeably higher in the treatment of 50, 100, or 200 μg/ml GFC‐F1, but it was significantly lower in the treatment of 25 μg/ml GFC‐F1 than in the LPS‐treated group (Figure 4b).

The pro‐inflammatory effects of two fractions on RAW 264.7 cells were further evaluated. GFC‐F1 at different concentrations (25, 50, 100, and 200 μg/ml) significantly induced the mRNA levels of *COX‐2*, *iNOS*, *TNF‐α*, and *IL‐1β* compared to the control (Figure 4c–f). Furthermore, the transcript levels of those genes continuously and significantly increased when treated with GFC‐F1 from 25 to 200 μg/ml. Although GFC‐F2 did not significantly change the mRNA levels of *COX‐2* (Figure 4c) and *IL‐1β* (Figure 4f) compared to the control, it significantly increased the mRNA levels of *iNOS* at the concentration from 50 to 200 μg/ml (Figure 4d), and the mRNA levels of *TNF‐α* at the concentration from 25 to 200 μg/ml (Figure 4e). In addition, the transcript levels of pro‐inflammatory cytokines in the GFC‐F2‐treated group were significantly lower than those in the GFC‐F1‐treated group.

**FIGURE 5:**
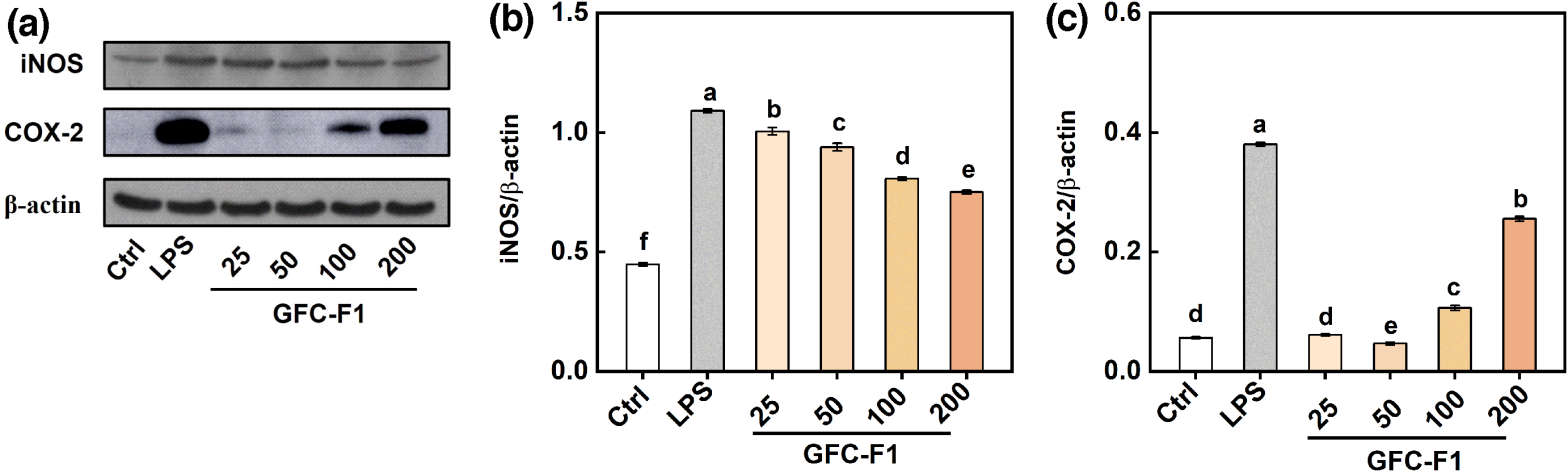
Effect of GFC‐F1 on COX‐2 and iNOS protein expression. After 18 h treatment of various concentrations of GFC‐F1 (25, 50, 100, and 200 μg/ml), the cells were harvested to determine the protein levels by Western blot analysis. COX‐2, cyclooxygenase‐2; GFC‐F1, gel filtration chromatography fraction 1; iNOS, inducible nitric oxide synthase; LPS, lipopolysaccharide.

Compared to the control, the iNOS protein level in RAW 264.7 cells treated with LPS and different concentrations of GFC‐F1 was significantly increased, but decreased with the increase in concentration of GFC‐F1 (Figure 5b). The COX‐2 protein level was clearly induced by LPS or GFC‐F1 at the concentration of 100 or 200 μg/ml, and increased with the increase of concentration of GFC‐F1 (Figure 5c).

### 3.4 GFC‐F1 activated NF‐κB and MAPK pathways in RAW 264.7 macrophages

**FIGURE 6:**
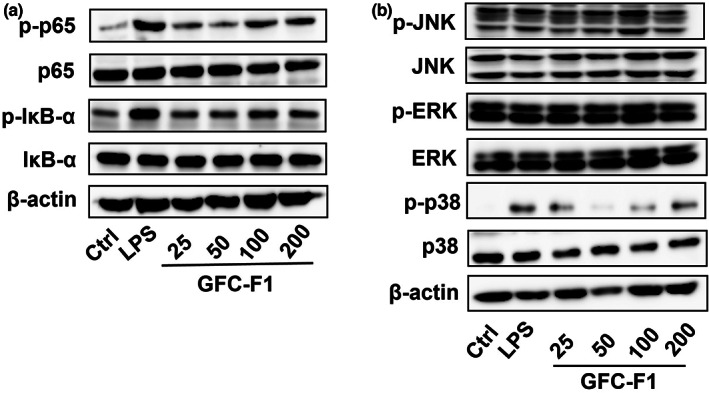
Effect of GFC‐F1 on the activation of NF‐κB (a) and MAPK (b) signaling pathways. After 18 h treatment of various concentrations of GFC‐F1 (25, 50, 100, and 200 μg/ml), the cells were harvested to determine the protein levels by Western blot analysis. GFC‐F1, gel filtration chromatography fraction 1; LPS, lipopolysaccharide; MAPK, mitogen‐activated protein kinase; NF‐κB, nuclear factor‐kappa B.

NF‐κB and MAPK pathways are the essential signaling pathways related to the inflammatory response. To determine if those pathways are responsible for GFC‐F1‐induced macrophage activation, immunoblot analysis was utilized to assess the phosphorylation state of crucial enzymes in NF‐κB and MAPK pathways. The transcription factor NF‐κB, consisting of the subunits p65 and p50, is involved in the transcriptional regulation of pro‐inflammatory kinases. NF‐κB activation requires the phosphorylation and degradation of its endogenous inhibitor (IκBα). Compared to the control, the protein levels of p‐p65 (the phosphorylation state of p65) were clearly increased, and the p‐IκB‐α (the phosphorylation state of IκB‐α (inhibitor of nuclear factor‐kappa B alpha)) protein levels were slightly increased by LPS or GFC‐F1 treatment; however, the p65 or IκB‐α protein levels were similar among treatments (Figure 6a). In addition to the NF‐κB transcription factor, MAP kinases (p38, JNK, and ERK) are involved in the production of cytokines and NO. It was found that the p‐p38 protein level was higher in LPS or GFC‐F1‐treated group than in the control; except for the p‐p38 protein level, the protein levels of other enzymes were similar among different treatments (Figure 6b).

**FIGURE 7:**
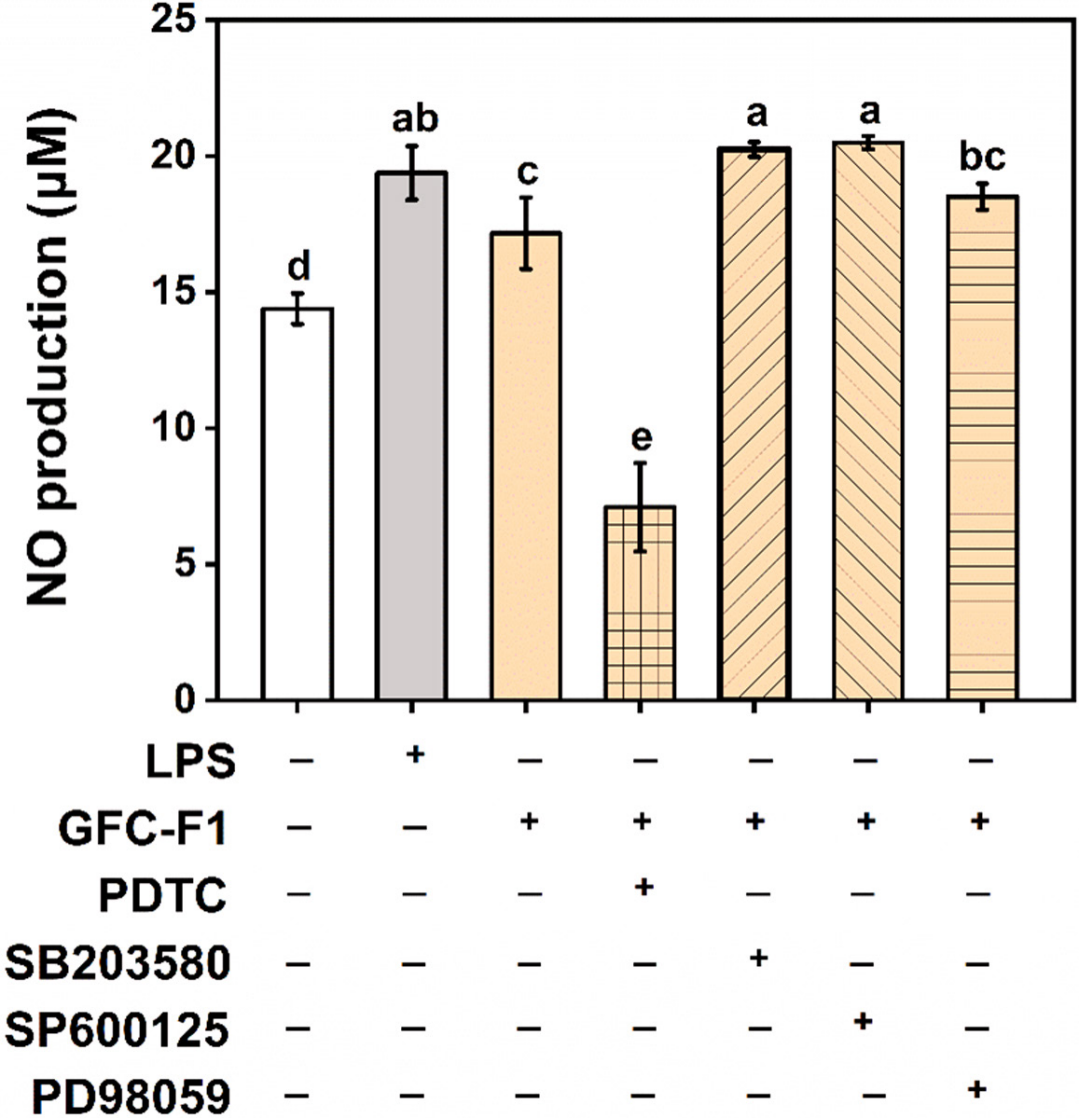
Effects of inhibitors, PDTC (NF‐κB inhibitor), SB203580 (p38 inhibitor), SP600125 (JNK inhibitor), and PD98059 (ERK inhibitor), on GFC‐F1‐induced NO production in RAW 264.7 cells. Cells were preincubated with inhibitors for 2 h and then treated with GFC‐F1 (200 μg/ml) for 12 h. The supernatants were used to detect NO production by Griess reagents. +/− means to treat the cells with (+) or without (−) the left sample or inhibitors. Data are presented as the mean ± SE (*n* = 3), and different lowercase letters on the bar indicate significantly different values (*p* < .05). GFC‐F1, gel filtration chromatography fraction 1; LPS, lipopolysaccharide; NO, nitric oxide; PDTC, pyrrolidine dithiocarbamate.

To provide further evidence of GFC‐F1‐activated NF‐κB and MAPK pathways, RAW 264.7 macrophages were pretreated with inhibitors of NF‐κB or MAPKs, and their effects on NO production in GFC‐F1‐treated RAW 264.7 cells were assessed. As illustrated in Figure 7, NO production was significantly increased in GFC‐F1‐treated RAW 264.7 cells compared to the control group; PDTC (pyrrolidine dithiocarbamate) (NF‐κB inhibitor) can efficiently inhibit NO production in GFC‐F1‐stimulated RAW 264.7 macrophages. In contrast, MAPK inhibitors, including SB203580 (p38 inhibitor), SP600125 (JNK inhibitor), and PD98059 (ERK inhibitor), did not reduce NO production but increased NO production.

## 4 DISCUSSION

*Panas ginseng* is the most well‐known traditional herbal medicine used as a therapeutic supplement or functional food to treat various diseases for a long time (Um et al., 2020). Previous studies have believed that the pharmacological effects of ginseng are derived from its various active components, mainly including ginsenosides, polysaccharides, peptides, etc. (Kim et al., 2017), and most researches mainly focus on the functions of ginsenosides and polysaccharides. Of them, ginsenosides, the major bioactive constituents of *P. ginseng*, show different therapeutic effects, including anti‐inflammatory, anticancer, anti‐oxidative, and antidiabetic effects (Kim, 2018). While ginseng polysaccharides, the most abundant components of ginseng, have antitumor, anti‐inflammatory, antioxidative, and immunomodulatory activities (Guo et al., 2021). Although *P. ginseng* is beneficial to human health, excessive intake of *P. ginseng* can still cause some side effects, such as “ginseng abuse syndrome” (GAS), which is a generally accepted adverse event related to *P. ginseng* (Siegel, 1979). A previous study has also reported that overconsumption of Korean red ginseng, the steamed root of *P. ginseng*, could induce “*shanghuo*” (Zhao et al., 2020).

There are similarities in symptoms between GAS and “*shanghuo*,” which are related to inflammation. It was reported that the water‐soluble substances in “heating fruits” may be the key components to induce inflammation. For example, the water‐soluble extracts of litchi, longan, and dried longan significantly increased the production of prostaglandin E2 (PGE_2_) and the expression of COX‐2 protein in RAW 264.7 macrophages (Huang & Wu, 2002). Moreover, water‐soluble extract of satsumas mandarin promoted pro‐inflammatory mediators' production, showing an apparent pro‐inflammatory effect (Yan et al., 2014). However, they found no obvious effects of EAE on PGE_2_ production (Huang & Wu, 2002; Yan et al., 2014). In this study, we also found that WSS significantly increased the expression levels of pro‐inflammatory cytokines (Figure 2b–d). What is more, it was HWSS that remarkedly enhanced the transcription level of pro‐inflammatory mediators (Figure 3b–d) rather than EAE and LWSS. Although EAE and LWSS had minimal effects on pro‐inflammatory mediators, it might be attributed to an emergency reaction of that fast response to eliminate adverse reaction of external stimulus, even slight stimuli by producing cytokines (Um et al., 2020).

In addition, we further purified two fractions (GFC‐F1 and GFC‐F2) from HWSS and found that GFC‐F1 induced a significant inflammation response (Figure 4b–f). Therefore, we could suggest that the high‐molecular water‐soluble substance of *P. ginseng*, GFC‐F1, plays a significant role in the pro‐inflammatory effect in the case of excessive ginseng consumption. Although the molecular weight of GFC‐F1 is 11–60 kDa with four primary components (Figure 1c), and some studies suggested that polysaccharides or proteins in the high‐molecular water‐soluble substances are the key components that cause inflammation (Li et al., 2010; Wang et al., 2016; Yan et al., 2014). For instance, the aqueous extract containing polysaccharides of North American ginseng has an immunostimulatory effect and can increase the production of NO, TNF‐α, and IL‐6 (Azike et al., 2015). Azike et al. (2015) also found that acidic polysaccharide fractions with molecular weight ≥ 100 kDa enhanced the production of NO and TNF‐α. There was also a study showing that acidic polysaccharide of Korean red ginseng activated macrophages through NF‐κB and AP‐1 as well as the enzymes of 255 signaling pathway, including ERK and JNK, thereby resulting in the activation of transcription factors (Byeon et al., 2012). Although a novel protein of American ginseng can dose‐dependently enhance NO production in murine peritoneal macrophages (Qi et al., 2016), there is currently no research to substantiate the pro‐inflammatory effect of proteins in *P. ginseng*. Therefore, the exact constituent of GFC‐F1 causing the occurrence of GAS needs further study in the future.

Inflammation, an immune response that protects our body against various stimuli, is closely associated with the release of pro‐inflammatory mediators such as iNOS, COX‐2, and pro‐inflammatory cytokines such as interleukins (ILs) and TNF‐α (Bak et al., 2012). The expression of pro‐inflammatory cytokines is regulated by the NF‐κB and MAPK pathways at both transcriptional and posttranscriptional levels (Bak et al., 2012; Choi et al., 2008). It is reported that thaumatin‐like protein isolated from *Litchi chinensis* has pro‐inflammatory activity and can enhance the expression of inflammation‐related genes (such as *COX‐2*, *iNOS*, *TNF‐α*, and *IL‐1β*) and *p65*; its pro‐inflammatory activity might be regulated by the NF‐κB pathway (Chen et al., 2020).

**FIGURE 8:**
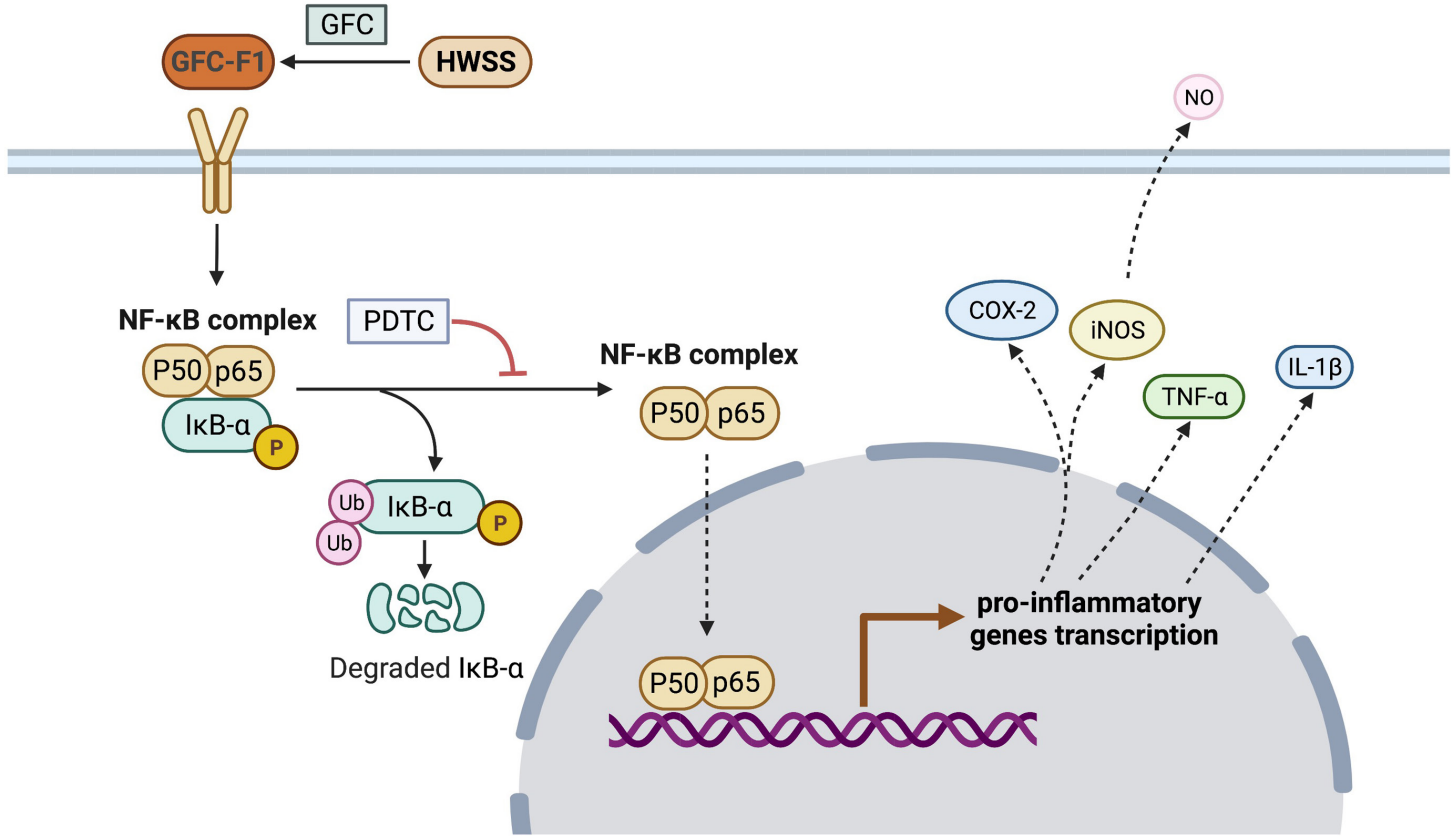
The signaling pathway underlying the *Panax ginseng* induced GAS. COX‐2, cyclooxygenase‐2; GFC‐F1, gel filtration chromatography fraction 1; HWSS, High‐molecular water‐soluble substances; IL‐1β, interleukin‐1β; iNOS, inducible nitric oxide synthase; NF‐κB, nuclear factor‐kappa B; PDTC, pyrrolidine dithiocarbamate; TNF‐α, tumor necrosis factor α. GFC‐F1 isolated from *P. ginseng* increases the expression of pro‐inflammatory genes through the activation of NF‐κB (p65 and IκBα) pathway in RAW 264.7 cells.

Furthermore, *P. ginseng* containing various pharmacological components has been well known as an immune modulator (Son et al., 2020), enhancing macrophage activity and producing many cytokines and inflammatory mediators, such as NO (Kang & Min, 2012). The aqueous extracts of *P. ginseng* could improve the expression of iNOS both at mRNA and protein levels by activating the NF‐κB pathway (Friedl et al., 2001). In addition, the water extract of wild‐simulated ginseng increased the production of pro‐inflammatory mediators (such as NO, iNOS, COX‐2, IL‐1β, IL‐6, and TNF‐α) and activated macrophages through MAPK, NF‐κB, and PI3K/AKT signaling pathways (Um et al., 2020). Herein, the present study found that WSS, HWSS isolated from WSS, and GFC‐F1 purified from HWSS enhanced the expression levels of pro‐inflammatory genes, such as *COX‐2*, *iNOS*, *TNF‐α*, and *IL‐1β* and NO production. Moreover, GFC‐F1 activated key factors in both NF‐κB and MAPK pathways in RAW 264.7 macrophages (Figure 6). However, NF‐κB inhibitor efficiently inhibited NO production in GFC‐F1‐stimulated RAW 264.7 macrophages, while MAPK inhibitors increased NO production rather than reducing NO production (Figure 7). Hence, we could conclude that the pro‐inflammatory activity of GFC‐F1 takes place through the regulation of the NF‐κB signaling pathway (including the phosphorylation of p65 and IκBα) rather than the MAPK pathway (Figure 8).

## 5 CONCLUSIONS

*Panas ginseng* can activate macrophages to enhance the immune system. However, excessive consumption of ginseng may cause overactivation of the immune system forming GAS. The present results suggested that GFC‐F1 (the molecular weight range of 11–60 kDa) purified from the high‐molecular water‐soluble substance of ginseng is the crucial component that caused GAS. Moreover, GFC‐F1 activates the NF‐κB pathway to trigger the immune system leading to inflammatory responses by inducing the phosphorylation of p65 and IκBα. These results provide a theoretical basis for improving ginseng quality and guidelines for the scientific consumption of ginseng. However, the specific component that may cause GAS still needs further study.

## CONFLICT OF INTEREST

The authors declare no conflict of interest.
